# Trends and disparities in inflammatory bowel disease and cardiovascular disease-related mortality in the United States from 1999 to 2023: A CDC WONDER analysis

**DOI:** 10.1016/j.ijcrp.2025.200438

**Published:** 2025-05-22

**Authors:** Syed Anjum Gardezi, Nakul Sachdeva, Insiya Mohammed Rampurawala, Akalanka Ranasinghe, Muhammad Umair Shehzad, Kieran Gill, Raheel Qureshi, Ashish Gupta, Ali Hasan, Muzammil Farhan, Azeem Hassan, Eeshal Zulfiqar, Mushood Ahmed, Raheel Ahmed

**Affiliations:** a Johns Hopkins Healthcare Aramco, Eastern Province, Saudi Arabia; b Department of Medicine, South Tyneside and Sunderland NHS Foundation Trust, Sunderland, UK; c Department of Medicine, Jagadguru Sri Shivarathreeshwara Medical College (JSSMC), Mysore, India; d Department of Cardiology, Queen Elizabeth Hospital Birmingham, Birmingham, UK; e Diabetes and Endocrinology, Western General Hospital, Edinburgh, UK; f Translational and Clinical Research Institute, Newcastle University Newcastle-upon-Tyne, UK; g Queen Elizabeth Hospital, Gateshead, UK; h Sunderland Royal Hospital, UK; i Department of Medicine, Imperial College, London, UK; j Department of Medicine, Dow University of Health Sciences, Karachi, Pakistan; k Department of Medicine, Rawalpindi Medical University, Rawalpindi, Pakistan; l National Heart & Lung Institute, Imperial College London, London, UK; m Department of Cardiology, Royal Brompton Hospital, London, UK

**Keywords:** Inflammatory bowel disease, Cardiovascular disease, CDC WONDER, Mortality

## Abstract

**Background:**

Individuals with inflammatory Bowel Disease (IBD) may face an increased risk of cardiovascular disease (CVD) due to chronic systemic inflammation and vascular dysfunction. While advancements in treatment have improved IBD management, its impact on cardiovascular mortality remains unclear. This study aims to analyze trends in IBD- and CVD-related mortality in the U.S. from 1999 to 2023, identifying high-risk populations based on age, sex, race, and geography.

**Methods:**

Mortality data for individuals aged 25 years and older from 1999 to 2023 were obtained from the CDC WONDER database. Crude mortality rates (CMRs) and age-adjusted mortality rates (AAMRs) per 100,000 persons were calculated. Temporal trends were assessed using Joinpoint regression analysis to estimate the annual percent change (APC) and average annual percent change (AAPC) in mortality rates.

**Results:**

Between 1999 and 2023, a total of 41,635 deaths were identified related to IBD and CVD among adults aged 25 years and older. The overall AAMR remained relatively stable from 1999 to 2018 before increasing sharply from 0.67 in 2018 to 1.03 in 2021 [APC: 15.63∗ (95 % CI: 11.66, 17.91); p = 0.0004], after which it plateaued through 2023. Males consistently exhibited higher AAMRs than females throughout the study period (Males: 1.10 vs. Females: 0.90 in 2023). When stratified by race, the highest AAMR was observed in NH White populations, followed by NH Black or African American individuals (1.21 vs. 0.64 in 2023). Regionally, the highest mortality was observed in the West, followed by the Midwest, the Northeast, and lastly, the South (AAMR of 1.02, 1.08, 0.87, and 0.97, respectively, in 2023). Rural areas (0.74) exhibited consistently higher AAMRs than urban areas (0.69) from 1999 to 2020. Mortality rates increased with age, with the highest burden observed in individuals aged 85 years and older.

**Conclusion:**

IBD- and CVD-related mortality has risen in the U.S., with the highest burden among males, NH White individuals, and older adults. Targeted interventions and enhanced cardiovascular screening are needed to reduce mortality in high-risk populations.

## 1 Introduction

Inflammatory Bowel Disease (IBD), comprising Crohn's disease and ulcerative colitis, is a common chronic inflammatory condition that affects both the gastrointestinal tract and other systems through extraintestinal manifestations [1]. Affecting approximately 4.9 million people in 2019, IBD continues to pose a significant global health burden, with the United States reporting the highest prevalence worldwide [2]. In the United States, IBD affects over 0.7 % of the population, with an estimated prevalence ranging from 2.4 to 3.1 million individuals [3,4]. The age- and sex-standardized incidence is 10.9 per 100,000 person-years, and projections suggest that by 2040, IBD-related age-standardized mortality rates will rise by 0.9 per 100,000 [3,5], highlighting the growing burden of this disease.

Patients with IBD are at an increased risk of developing cardiovascular diseases including atherosclerosis, myocardial infarction and venous thrombosis [6,7]. This association is mainly attributed to chronic systemic inflammation. Elevated levels of inflammatory cytokines, C-reactive protein (CRP), and homocysteine in IBD contribute to vascular injury and atherogenesis, while gut microbiota dysbiosis and increased coagulation factors may further predispose patients to arterial thromboembolic events, independent of traditional cardiovascular risk factors [7,8]. Notably, despite this elevated cardiovascular risk, conventional risk factors such as hypertension and hypercholesterolemia are less prevalent in IBD patients, suggesting inflammation plays a more significant role [8,9].

Despite the known association between IBD and cardiovascular disease (CVD), national mortality trends in this population remain unclear. The uneven distribution of disease burden across populations and regions complicates risk assessment, reinforcing the importance of an in-depth analysis. To address this gap, we utilize the CDC WONDER database to analyze IBD- and cardiovascular disease-related mortality trends from 1999 to 2023, stratified by age, sex, race, and geographic location, to identify high-risk subgroups and evolving disease patterns. Understanding these trends can help guide targeted interventions and improve outcomes in this population.

## 2 Methods

### 2.1 Study setting and population

Mortality data related to IBD and CVD in the United States were obtained from the Centers for Disease Control and Prevention Wide-Ranging Online Data for Epidemiologic Research (CDC WONDER) database [10]. CDC-WONDER is an exhaustive repository of death certificate data from the fifty states of the USA as well as the District of Columbia. We utilized the Multiple Cause-of-Death Public Use Files to identify cases in which IBD or CVD was recorded as either an underlying or contributing cause of death. Patients were identified using the International Classification of Diseases 10th Revision Clinical Modification (ICD-10-CM) codes K50-51 for IBD and I00-I99 for CVD in individuals ≥25 years of age. These codes have been used by other researchers in the past [11,12]. As the dataset is publicly available and fully de-identified, institutional review board approval was not required. The study was conducted in accordance with the Strengthening the Reporting of Observational Studies in Epidemiology (STROBE) guidelines [13].

### 2.2 Data abstraction

We extracted data on IBD- and CVD-related mortality along with corresponding population estimates, demographics (age, sex, race/ethnicity), and geographic information (state and urban–rural classification) for the period 1999 to 2023. Race and ethnicity were categorized as non-Hispanic (NH) White, NH Black or African American, NH Other (including NH Asian or Pacific Islander, NH American Indian or Alaska Native), and Hispanic or Latino—consistent with classifications used in prior CDC WONDER analyses based on information reported on death certificates. Age was stratified into the following categories: 25–34, 35–44, 45–54, 55–64, 65–74, 75–84, and ≥85 years. Mortality trends were further assessed across individual states, U.S. Census regions (Northeast, Midwest, South, and West), and by urban–rural status at the county level. Counties were designated as rural (micropolitan and noncore areas) or urban (large central metro, large fringe metro, medium metro, small metro) according to the 2013 National Center for Health Statistics Urban–Rural Classification Scheme [14]. These stratifications have been used in prior research as well [[15], [16], [17], [18]].

### 2.3 Statistical analysis

Crude and age-adjusted mortality rates per 100,000 population were determined. Crude mortality rates (CMRs) were derived by dividing the annual number of IBD- and CVD-related deaths by the corresponding U.S. population for each year. AAMRs were computed by standardizing mortality figures to the 2000 U S. standard population, as previously described [19]. The Joinpoint Regression Program (Joinpoint V 5.2.0.0, National Cancer Institute) was used to determine trends in AAMRs and CMRs using annual percent change (APC) [20]. This method identifies significant changes in AAMRs and CMRs over time by fitting log-linear regression models where temporal variation occurred. The APCs and its 95 % confidence intervals (CI)were estimated for each identified segment, using the Monte Carlo permutation method for statistical inference. Trends were considered increasing or decreasing if the slope of the regression differed significantly from zero, determined by 2-tailed t testing. Statistical significance was set at p < 0.05. Additionally, we conducted a sensitivity analysis, where IBD was listed as the underlying cause of death, and CVD was listed as the contributing cause of death and vice versa.

## 3 Results

### 3.1 Annual trends

**Fig. 1:**
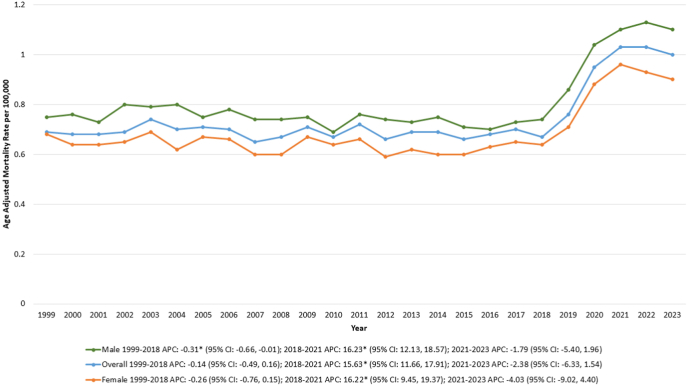
Overall and sex-stratified inflammatory bowel disease and cardiovascular disease-related age-adjusted mortality rates (AAMRs) per 100,000 individuals in the United States, 1999 to 2023.

Between 1999 and 2023, a total of 41,635 deaths were recorded among adults (≥25 years) due to IBD and CVD. The AAMR remained relatively stable from 1999 to 2018, before rising sharply from 0.67 in 2018 to 1.03 in 2021 [APC: 15.63∗ (95 % CI: 11.66, 17.91); p = 0.0004]. After 2021, the AAMR plateaued, showing no significant change through 2023 (Table S1, Table S2, Table S3, Fig. 1).

### 3.2 IBD and CVD-related AAMR stratified by sex

Throughout the study period, the AAMR was consistently higher in males than in females.

Among males, the AAMR declined slightly from 0.75 in 1999 to 0.74 in 2018 [APC: −0.31∗ (95 % CI: −0.66, −0.01); p = 0.04], followed by a sharp increase to 1.10 in 2021 [APC: 16.23∗ (95 % CI: 12.13, 18.57); p < 0.000001], after which it remained stable through 2023.

In females, the AAMR remained steady from 1999 to 2018, reaching 0.64 in 2018 before rising to 0.96 in 2021 [APC: 16.22∗ (95 % CI: 9.45, 19.37); p = 0.03]. The rate then stabilized through 2023 (Table S2, Table S3, Fig. 1).

### 3.3 IBD and CVD-related AAMR stratified race/ethnicity

Over the study period, the highest AAMR was observed in NH White populations, followed by NH Black or African American individuals.

Among NH White individuals, the AAMR remained stable from 1999 to 2018 before rising sharply from 0.82 in 2018 to 1.24 in 2021 [APC: 16.02∗ (95 % CI: 10.50, 18.52); p = 0.02], after which it remained relatively stable through 2023.

**Fig. 2:**
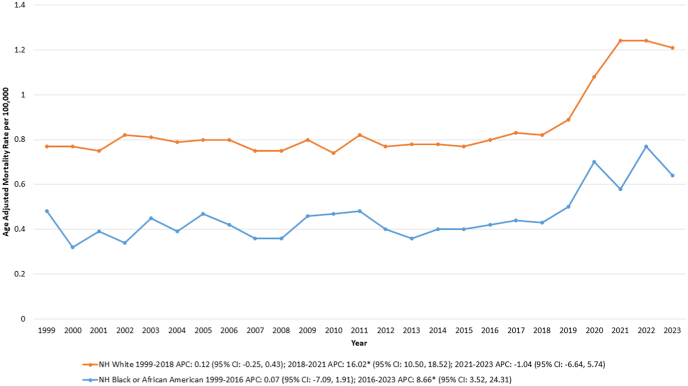
Inflammatory bowel disease and cardiovascular disease-related age-adjusted mortality rates (AAMRs) per 100,000 individuals stratified by race/ethnicity in the United States, 1999 to 2023.

Similarly, in NH Black individuals, the AAMR was stable from 1999 to 2016, followed by a significant increase from 0.42 in 2016 to 0.64 in 2023 [APC: 8.66∗ (95 % CI: 3.52, 24.31); p = 0.004] (Table S2, Table S4, Fig. 2).

### 3.4 IBD and CVD-related AAMR stratified by geographical region

#### 3.4.1 Statewide

Throughout the study period, significant statewide variation in IBD and CVD-related mortality was observed. Between 1999 and 2020, Rhode Island exhibited the highest AAMR of 1.21 while Hawaii reflected the lowest AAMR of 0.27. States falling within the top 90th percentile for mortality rates between 1999 and 2020 included Rhode Island, Oregon, Vermont, Nebraska and Oklahoma. In contrast, Hawaii, Louisiana, District of Columbia, New Mexico and Delaware exhibited some of the lowest mortality rates, corresponding to the lower 10th percentile during this period.

In the subsequent period from 2021 to 2023, the states with the highest mortality rates were Oregon, Nebraska, Oklahoma, Wyoming and Idaho, whereas Alaska, North Dakota, Alabama, Connecticut and Delaware ranked in the lowest 10th percentile (Table S5).

##### 3.4.1.1 Census region

From 1999 to 2023, the highest IBD and CVD-related mortality rates were observed in the Western region, followed by the Midwest, the Northeast and lastly, the Southern region.

The AAMR in the Western and Midwest regions remained relatively stable throughout the study period. In the Northeast, the AAMR declined from 0.89 in 1999 to 0.65 in 2015 [APC: −1.49∗ (95 % CI: −3.06, −0.49); p = 0.002], followed by a significant increase, reaching 0.87 in 2023 [APC: 5.13∗ (95 % CI: 2.71, 11.46); p < 0.000001].

**Fig. 3:**
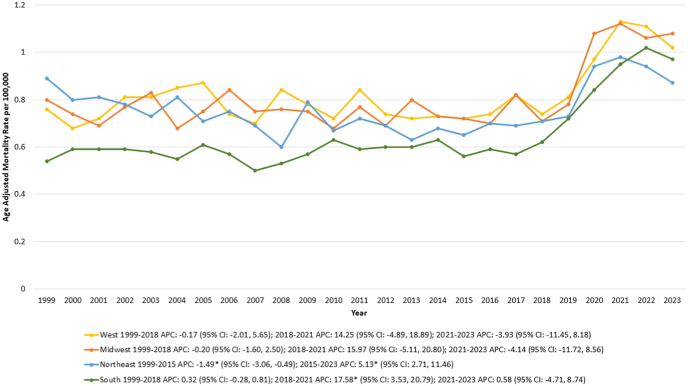
Inflammatory bowel disease and cardiovascular disease-related age-adjusted mortality rates (AAMRs) per 100,000 individuals stratified by census region in the United States, 1999 to 2023.

Similarly, in the South, the AAMR remained stable from 1999 to 2018 before rising significantly from 0.62 in 2018 to 0.95 in 2021 [APC: 17.58∗ (95 % CI: 3.53, 20.79); p = 0.03], after which it remained stable through 2023 (Table S6, Fig. 3).

##### 3.4.1.2 Urban-rural

Between 1999 and 2020, AAMRs related to IBD and CVD were consistently higher in rural regions compared to urban counterparts. In rural areas, the AAMR exhibited a gradual increase from 0.66 in 1999 to 0.78 in 2018 [APC: 0.92∗ (95 % CI: 0.24, 1.44); p = 0.02] followed by a sharp rise to 1.09 in 2020 [APC: 15.90∗ (95 % CI: 5.88, 20.93); p < 0.000001].

**Fig. 4:**
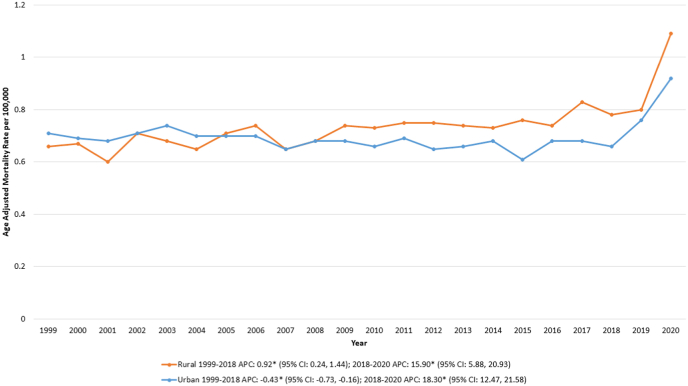
Inflammatory bowel disease and cardiovascular disease-related age-adjusted mortality rates (AAMRs) per 100,000 individuals stratified by urbanization in the United States, 1999 to 2020 **∗** Data for urbanization AAMRs was unavailable for 2021–2023.

Conversely, urban areas demonstrated a decline in AAMR from 0.71 in 1999 to 0.66 in 2018 [APC: −0.43∗ (95 % CI: −0.73, −0.16); p = 0.001], before experiencing a sharp increase to 0.92 in 2020 [APC: 18.30∗ (95 % CI: 12.47, 21.58); p < 0.000001] (Table S7, Fig. 4).

### 3.5 IBD and CVD-related AAMR stratified by ten year age groups

**Fig. 5:**
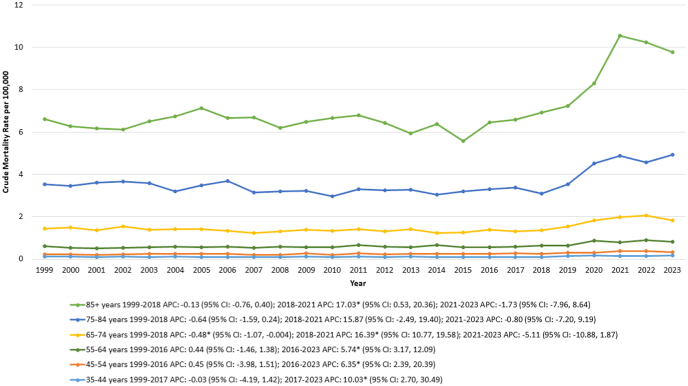
Crude mortality rates (CMRs) per 100,000 individuals stratified by age groups in the United States, 1999 to 2023.

When the analysis was stratified by age groups, the highest CMRs were identified in the 85+ age categories, followed by the 75–84, 65–74, 55–64, and 45–54 age groups. Conversely, the 35–44 age group exhibited the lowest CMRs (Fig. 5).

### 3.6 Results of the sensitivity analysis

When IBD was designated as the underlying cause of death (UCD) and CVD as a multiple/contributing cause of death (MCD), a total of 7,828 deaths were recorded between 1999 and 2020, and 1,510 deaths occurred from 2021 to 2023 (Table S8).

Conversely, when CVD was the underlying cause and IBD a contributing factor, there were 11,848 deaths from 1999 to 2020 and 2,307 deaths from 2021 to 2023 (Table S9).

## 4 Discussion

This retrospective analysis of mortality data from 1999 to 2023 reveals several key findings into IBD and CVD-related deaths. First, the overall AAMR remained stable until 2018, followed by a sharp increase through 2021, after which it plateaued. Males consistently exhibited higher AAMRs than females. Racial disparities were evident, with NH White populations demonstrating the highest AAMRs. Geographic variations were also observed, with the highest mortality rates occurring in the Western region, while states in the top 90th percentile had more than three times the AAMR of those in the bottom 10th percentile. Rural areas consistently exhibited higher AAMRs than urban areas from 1999 to 2020. Finally, an age-related increase in mortality rates was observed, with the highest burden observed in individuals aged 85 years and older.

The trend of stable mortality rates from 1999 to 2018, followed by a marked rise after 2018, is consistent with previous research on inflammatory bowel disease-related mortality [21]. IBD has been associated with an elevated risk of cardiovascular diseases, including cerebrovascular disease, coronary heart disease and myocardial infarction [7,22]. Chronic systemic inflammation in IBD plays a crucial role in atherosclerosis progression, contributing to CVD risk by promoting endothelial dysfunction, oxidative stress, and macrophage accumulation through elevated markers such as CRP [9,23]. Additionally, metabolic disturbances, such as dyslipidemia, further exacerbate this risk. A study by Koutroumpakis et al. found that patients with IBD had higher triglyceride levels and lower high-density lipoprotein (HDL) levels compared to healthy controls, both of which are established risk factors for CVD [[24], [25], [26]]. Moreover, medications used in IBD treatment may contribute to CVD risk, with JAK inhibitors linked to hypertension and altered lipid profiles, while prolonged corticosteroid use is linked to increased risk of major adverse cardiovascular events [27,28]. The COVID-19 pandemic has also impacted this population, as both IBD and COVID-19 can induce hyperinflammatory states, potentially compounding CVD risk [29,30]. The observed rise in mortality rates after 2018 through 2021 can also be explained by the pandemic's impact, as it placed significant strain on healthcare systems, delayed routine medical care, and a disproportionately severe impact on individuals with underlying conditions such as IBD and CVD [31,32].

Throughout the study period, sex-based differences remained evident, as males consistently exhibited higher AAMRs compared to females. While prior research suggests a male predominance in ulcerative colitis later in life and a higher incidence of Crohn's disease in females [33], the mortality burden does not necessarily follow the same pattern. The higher AAMRs in males may be influenced by factors other than disease incidence, including a more aggressive disease course, higher rates of surgical interventions, increased smoking prevalence, a greater likelihood of disease-related complications and an increased risk of colorectal cancer [34,35]. Similarly, a meta-analysis by Salem et al. indicated that males with IBD face higher risks of early mortality, whereas females experience greater levels of anxiety and depression [36]. Additionally, males often present with more extensive disease at diagnosis, including widespread colitis, which increases the likelihood of hospitalization, corticosteroid refractoriness, and the need for surgical intervention such as colectomy [37]. The more complicated disease course in males may also be linked to sex hormone influences on immune activation and intestinal physiology, as well as environmental factors like smoking and diet, both of which are more prevalent in males and associated with worse disease outcomes [38,39]. Moreover, males tend to be older at the time of diagnosis and may have a higher burden of comorbidities, particularly cardiovascular disease, which further contributes to increased mortality risk [36].

Conversely, females may benefit from protective factors such as estrogen's anti-inflammatory properties, which can modulate immune responses and possibly reduce the severity of inflammation. For instance, upregulation of G protein-coupled estrogen receptor (GPER) and estrogen receptor α (ERα) in IBD patients suggests a protective role of estrogen signaling in maintaining epithelial homeostasis and regulating intestinal inflammation [40]. However, hormonal influences in females are complex, as oral contraceptive (OCP) use and hormone replacement therapy (HRT) have been associated with an increased risk of IBD, potentially counteracting some of the protective effects of estrogen [41,42]. Additionally, sex-based differences in healthcare utilization may also contribute to variations in disease outcomes, particularly in the higher surgical rates observed in males [43]. Females, on the other hand, are are generally less inclined to undergo surgical interventions such as colectomy due to concerns about postoperative complications, fertility implications, and body image [44]. Nonetheless, IBD in females may be underdiagnosed or misdiagnosed due to differences in symptom presentation and diagnostic biases, potentially leading to an underestimation of IBD-related mortality in women [45].

We found significant racial/ethnic disparities in IBD and CVD-related mortality, with the highest rates observed among NH White individuals. This finding is consistent with previous studies indicating a higher prevalence of IBD in this population [21,46]. However, while the overall disease burden of IBD is lower in ethnic minorities compared to NH Whites, IBD-related hospitalizations and deaths appear disproportionately high among NH Black individuals [46]. This pattern aligns with our findings, where NH White individuals had the highest mortality rates, followed by NH Black individuals. The higher incidence of IBD in NH White populations may be attributed to a combination of genetic predisposition, environmental exposures, and lifestyle factors. Genetic susceptibility, particularly mutations in the NOD2 gene, is more prevalent in individuals of European descent, increasing their risk of developing IBD [47,48]. Moreover, the hygiene hypothesis suggests that diminished microbial exposure during early life, more common in industrialized settings, may impair immune system regulation, contributing to disease development [49]. While the overall prevalence of IBD is lower in NH Black individuals, studies have shown that those who do have the disease are more likely to require hospitalization [46,50]. This disparity may result from more severe disease presentations, including a higher prevalence of perianal involvement and fistulas among Black patients, which necessitate increased medical interventions. Additionally, Black patients are less likely to receive regular care from gastroenterology specialists, leading to increased emergency department visits and hospitalizations [[50], [51], [52]]. Moreover, NH Black patients undergoing surgery for IBD have higher rates of postoperative complications, including infections and longer hospital stays, which may contribute to the increased hospitalization rates [53]. Furthermore, socioeconomic factors and disparities in healthcare access contribute to delays in diagnosis and treatment, exacerbating disease severity and increasing the likelihood of hospitalization.

We observed higher IBD-related mortality rates in rural areas compared to urban regions. This finding contrasts with some earlier studies that reported higher IBD prevalence in urban settings [54]. Rural residents often face significant barriers to healthcare access, such as fewer healthcare facilities and providers, leading to delayed diagnoses and suboptimal management of chronic conditions like IBD [55]. Additionally, socioeconomic challenges, such as higher poverty rates and lower educational attainment, prevalent in rural communities, can limit access to healthcare and resources necessary for effective disease management, further contributing to higher mortality rates. Moreover, urban patients may have better insurance coverage and financial means to afford advanced treatments, which are less accessible in rural settings, potentially exacerbating disparities in disease outcomes [56]. Furthermore, we found that the West region had the highest mortality. These regional disparities may be influenced by factors such as differences in environmental exposures, healthcare infrastructure, and population demographics. Future research should aim to identify the underlying factors driving these variations to improve outcomes.

The findings of this study have significant clinical and public health implications. Clinically, the demonstrated increase in IBD- and CVD-related mortality, particularly among specific high-risk populations, emphasizes the need for increased cardiovascular risk screening and proactive management in patients with IBD. Early identification and intervention could mitigate adverse outcomes in these vulnerable groups. From a public health standpoint, understanding how mortality differs across age, sex, race, and geographic groups allows for more precise targeting of resources and the design of prevention efforts that address the needs of those most at risk. These findings highlight the importance of multidisciplinary care to reduce cardiovascular mortality and improve health equity in patients with IBD.

### 4.1 Limitations

This study is subject to several limitations. Primarily, the use of death certificate data may lead to misclassification of deaths attributable to IBD- and CVD. Additionally, the absence of clinical details, such as disease severity, treatment history, and the presence of extraintestinal manifestations, limits our ability to fully contextualize individual mortality outcomes. Furthermore, the database lacks individual-level factors such as socioeconomic status, healthcare access, lifestyle behaviors, and comorbidities such as hypertension and dyslipidemia, which are important contributors to cardiovascular risk. While age adjustment was performed, it does not fully account for evolving trends in IBD management, including advancements in biologic therapies and disparities in healthcare utilization over time. Lastly, variations in diagnostic coding and reporting practices across regions may impact mortality estimates, warranting cautious interpretation of the findings.

## 5 Conclusion

Over the study period, IBD and CVD-related mortality remained stable until 2018, followed by a sharp increase in 2021 before stabilizing. Males, NH White individuals, and older adults experienced the highest mortality burden. Mortality rates were highest in the Western region, with rural areas showing greater mortality than urban areas. These findings highlight the need for targeted interventions, improved disease management, and equitable healthcare access to address disparities and reduce mortality.

## CRediT authorship contribution statement

**Syed Anjum Gardezi:** Writing – original draft, Visualization, Project administration, Conceptualization. **Nakul Sachdeva:** Writing – original draft, Resources, Investigation. **Insiya Mohammed Rampurawala:** Writing – original draft. **Akalanka Ranasinghe:** Writing – original draft, Project administration. **Muhammad Umair Shehzad:** Writing – original draft, Software, Resources. **Kieran Gill:** Writing – original draft, Project administration. **Raheel Qureshi:** Writing – original draft, Formal analysis, Data curation. **Ashish Gupta:** Writing – original draft, Visualization, Validation. **Ali Hasan:** Software, Methodology. **Muzammil Farhan:** Software, Methodology. **Azeem Hassan:** Writing – original draft, Software. **Eeshal Zulfiqar:** Writing – original draft, Supervision, Methodology, Formal analysis. **Mushood Ahmed:** Writing – review & editing, Visualization, Validation, Supervision, Conceptualization. **Raheel Ahmed:** Writing – review & editing, Visualization, Validation, Project administration.

## Ethical approval

No ethical approval was required for the study.

## Consent

No consent was needed.

## Data availability statement

All data generated or analyzed during this study are included in this article. Further inquiries can be directed to the corresponding author.

## Financial support

No financial support was received for the study.

## Declaration of competing interest

The authors declare no conflicts of interest.
