# Supplementary material for: Trends and disparities in inflammatory bowel disease and cardiovascular disease-related mortality in the United States from 1999 to 2023: A CDC WONDER analysis
Source: Int J Cardiol Cardiovasc Risk Prev. 2025 May 22;26:200438. doi: 10.1016/j.ijcrp.2025.200438 (PMC12159954; doi:10.1016/j.ijcrp.2025.200438)
Supplement: Multimedia component 1 [file mmc1.docx]

**Supplementary Appendix**

**Supplemental Table 1: Inflammatory bowel disease (IBD) and cardiovascular disease (CVD)-related deaths, stratified by Sex and Race in the United States, 1999 to 2023**

| Year | Overall | Female | Male | NH Black or African American | NH White | Hispanic or Latino | NH Others |
| --- | --- | --- | --- | --- | --- | --- | --- |
| 1999 | 1273 | 715 | 558 | 79 | 1163 | 22 | - |
| 2000 | 1245 | 694 | 551 | 51 | 1156 | 25 | 10 |
| 2001 | 1238 | 693 | 545 | 65 | 1144 | 16 | 11 |
| 2002 | 1320 | 733 | 587 | 62 | 1218 | 25 | 12 |
| 2003 | 1342 | 741 | 601 | 77 | 1232 | 19 | 11 |
| 2004 | 1340 | 712 | 628 | 71 | 1229 | 32 | - |
| 2005 | 1390 | 784 | 606 | 81 | 1259 | 35 | 12 |
| 2006 | 1411 | 771 | 640 | 72 | 1297 | 29 | 11 |
| 2007 | 1313 | 696 | 617 | 67 | 1208 | 26 | 10 |
| 2008 | 1356 | 720 | 636 | 71 | 1240 | 36 | - |
| 2009 | 1461 | 824 | 637 | 90 | 1315 | 36 | 18 |
| 2010 | 1395 | 771 | 624 | 93 | 1250 | 39 | 11 |
| 2011 | 1570 | 850 | 720 | 93 | 1408 | 48 | 19 |
| 2012 | 1508 | 796 | 712 | 91 | 1365 | 40 | 11 |
| 2013 | 1547 | 827 | 720 | 82 | 1401 | 39 | 21 |
| 2014 | 1577 | 825 | 752 | 96 | 1409 | 47 | 23 |
| 2015 | 1548 | 813 | 735 | 96 | 1386 | 51 | 12 |
| 2016 | 1666 | 889 | 777 | 101 | 1496 | 44 | 22 |
| 2017 | 1739 | 938 | 801 | 111 | 1546 | 49 | 32 |
| 2018 | 1773 | 939 | 834 | 111 | 1573 | 55 | 28 |
| 2019 | 1993 | 1017 | 976 | 137 | 1753 | 70 | 32 |
| 2020 | 2481 | 1273 | 1208 | 203 | 2143 | 85 | 45 |
| 2021 | 2663 | 1397 | 1266 | 156 | 2335 | 116 | 36 |
| 2022 | 2777 | 1433 | 1344 | 204 | 2434 | 76 | 47 |
| 2023 | 2709 | 1388 | 1321 | 186 | 2378 | 93 | 36 |

NH, Non-Hispanic

**Supplemental Table 2: Annual percent change (APC) of IBD and CVD-related age-adjusted mortality rates per 100,000 in the United States, 1999 to 2023**

| Year Interval | APC (95% confidence interval) |
| --- | --- |
| **Overall** |  |
| 1999-2018 | -0.14 (-0.49, 0.16) |
| 2018-2021 | 15.63* (11.66, 17.91) |
| 2021-2023 | -2.38 (-6.33, 1.54) |
| **Sex** |  |
| **Female** |  |
| 1999-2018 | -0.26 (-0.76, 0.15) |
| 2018-2021 | 16.22* (9.45, 19.37) |
| 2021-2023 | -4.03 (-9.02, 4.40) |
| **Male** |  |
| 1999-2018 | -0.31* (-0.66, -0.01) |
| 2018-2021 | 16.23* (12.13, 18.57) |
| 2021-2023 | -1.79 (-5.40, 1.96) |
| **Race** |  |
| **NH Black or African American** |  |
| 1999-2016 | 0.07 (-7.09, 1.91) |
| 2016-2023 | 8.66* (3.52, 24.31) |
| **NH White** |  |
| 1999-2018 | 0.12 (-0.25, 0.43) |
| 2018-2021 | 16.02* (10.50, 18.52) |
| 2021-2023 | -1.04 (-6.64, 5.74) |
| **Census region** |  |
| **Northeast** |  |
| 1999-2015 | -1.49* (-3.06, -0.49) |
| 2015-2023 | 5.13* (2.71, 11.46) |
| **Midwest** |  |
| 1999-2018 | -0.20 (-1.60, 2.50) |
| 2018-2021 | 15.97 (-5.11, 20.80) |
| 2021-2023 | -4.14 (-11.72, 8.56) |
| **South** |  |
| 1999-2018 | 0.32 (-0.28, 0.81) |
| 2018-2021 | 17.58* (3.53, 20.79) |
| 2021-2023 | 0.58 (-4.71, 8.74) |
| **West** |  |
| 1999-2018 | -0.17 (-2.01, 5.65) |
| 2018-2021 | 14.25 (-4.89, 18.89) |
| 2021-2023 | -3.93 (-11.45, 8.18) |
| **Urbanization** |  |
| **Urban** |  |
| 1999-2018 | -0.43* (-0.73, -0.16) |
| 2018-2020 | 18.30* (12.47, 21.58) |
| **Rural** |  |
| 1999-2018 | 0.92* (0.24, 1.44) |
| 2018-2020 | 15.90* (5.88, 20.93) |
| **Ten-year age groups**** |  |
| **35-44 years** |  |
| 1999-2017 | -0.03 (-4.19, 1.42) |
| 2017-2023 | 10.03* (2.70, 30.49) |
| **45-54 years** |  |
| 1999-2016 | 0.45 (-3.98, 1.51) |
| 2016-2023 | 6.35* (2.39, 20.39) |
| **55-64 years** |  |
| 1999-2016 | 0.44 (-1.46, 1.38) |
| 2016-2023 | 5.74* (3.17, 12.09) |
| **65-74 years** |  |
| 1999-2018 | -0.48* (-1.07, -0.004) |
| 2018-2021 | 16.39* (10.77, 19.58) |
| 2021-2023 | -5.11 (-10.88, 1.87) |
| **75-84 years** |  |
| 1999-2018 | -0.64 (-1.59, 0.24) |
| 2018-2021 | 15.87 (-2.49, 19.40) |
| 2021-2023 | -0.80 (-7.20, 9.19) |
| **85+ years** |  |
| 1999-2018 | -0.13 (-0.76, 0.40) |
| 2018-2021 | 17.03* (0.53, 20.36) |
| 2021-2023 | -1.73 (-7.96, 8.64) |

* Significant values

** For age groups, crude mortality rates were used for calculating APCs

**Supplemental Table 3: Overall and Sex‐Stratified** **IBD and CVD-related Age-Adjusted Mortality Rates per 100,000 in the United States, 1999 to 2023**

|  | Age-Adjusted Rate (95% confidence interval) | | |
| --- | --- | --- | --- |
| Year | **Overall** | **Female** | **Male** |
| 1999 | 0.69 (0.65 - 0.73) | 0.68 (0.63 - 0.73) | 0.75 (0.69 - 0.82) |
| 2000 | 0.68 (0.64 - 0.72) | 0.64 (0.59 - 0.68) | 0.76 (0.7 - 0.82) |
| 2001 | 0.68 (0.64 - 0.71) | 0.64 (0.59 - 0.68) | 0.73 (0.67 - 0.79) |
| 2002 | 0.69 (0.65 - 0.73) | 0.65 (0.61 - 0.7) | 0.8 (0.73 - 0.86) |
| 2003 | 0.74 (0.7 - 0.78) | 0.69 (0.64 - 0.74) | 0.79 (0.72 - 0.85) |
| 2004 | 0.7 (0.66 - 0.73) | 0.62 (0.57 - 0.67) | 0.8 (0.73 - 0.86) |
| 2005 | 0.71 (0.67 - 0.75) | 0.67 (0.62 - 0.71) | 0.75 (0.69 - 0.82) |
| 2006 | 0.7 (0.66 - 0.73) | 0.66 (0.62 - 0.71) | 0.78 (0.72 - 0.84) |
| 2007 | 0.65 (0.62 - 0.69) | 0.6 (0.55 - 0.64) | 0.74 (0.68 - 0.8) |
| 2008 | 0.67 (0.64 - 0.71) | 0.6 (0.55 - 0.64) | 0.74 (0.68 - 0.79) |
| 2009 | 0.71 (0.67 - 0.75) | 0.67 (0.62 - 0.72) | 0.75 (0.69 - 0.81) |
| 2010 | 0.67 (0.63 - 0.71) | 0.64 (0.59 - 0.69) | 0.69 (0.64 - 0.75) |
| 2011 | 0.72 (0.69 - 0.76) | 0.66 (0.62 - 0.71) | 0.76 (0.7 - 0.81) |
| 2012 | 0.66 (0.62 - 0.69) | 0.59 (0.55 - 0.63) | 0.74 (0.69 - 0.8) |
| 2013 | 0.69 (0.65 - 0.72) | 0.62 (0.58 - 0.66) | 0.73 (0.68 - 0.79) |
| 2014 | 0.69 (0.66 - 0.73) | 0.6 (0.56 - 0.65) | 0.75 (0.7 - 0.81) |
| 2015 | 0.66 (0.62 - 0.69) | 0.6 (0.55 - 0.64) | 0.71 (0.66 - 0.76) |
| 2016 | 0.68 (0.64 - 0.71) | 0.63 (0.59 - 0.68) | 0.7 (0.65 - 0.75) |
| 2017 | 0.7 (0.66 - 0.73) | 0.65 (0.61 - 0.69) | 0.73 (0.68 - 0.78) |
| 2018 | 0.67 (0.64 - 0.7) | 0.64 (0.59 - 0.68) | 0.74 (0.69 - 0.8) |
| 2019 | 0.76 (0.73 - 0.8) | 0.71 (0.67 - 0.76) | 0.86 (0.8 - 0.92) |
| 2020 | 0.95 (0.91 - 0.99) | 0.88 (0.83 - 0.93) | 1.04 (0.98 - 1.1) |
| 2021 | 1.03 (0.99 - 1.07) | 0.96 (0.91 - 1.02) | 1.1 (1.04 - 1.16) |
| 2022 | 1.03 (0.99 - 1.07) | 0.93 (0.88 - 0.98) | 1.13 (1.07 - 1.2) |
| 2023 | 1 (0.96 - 1.04) | 0.9 (0.85 - 0.95) | 1.1 (1.04 - 1.16) |

**Supplemental Table 4: IBD and CVD-related Age-Adjusted Mortality Rates per 100,000, Stratified by Race in the United States, 1999 to 2023**

|  | Age-Adjusted Rate (95% confidence interval) | | | |
| --- | --- | --- | --- | --- |
| Year | **NH Black or  African American** | **NH White** | **Hispanic or  Latino** | **NH others** |
| 1999 | 0.48 (0.38 - 0.6) | 0.77 (0.73 - 0.81) | 0.22 (0.13 - 0.35) | - |
| 2000 | 0.32 (0.24 - 0.42) | 0.77 (0.73 - 0.82) | 0.3 (0.19 - 0.45) | Unreliable |
| 2001 | 0.39 (0.29 - 0.5) | 0.75 (0.71 - 0.79) | Unreliable | Unreliable |
| 2002 | 0.34 (0.25 - 0.44) | 0.82 (0.77 - 0.87) | 0.26 (0.17 - 0.39) | Unreliable |
| 2003 | 0.45 (0.35 - 0.56) | 0.81 (0.77 - 0.86) | Unreliable | Unreliable |
| 2004 | 0.39 (0.3 - 0.49) | 0.79 (0.75 - 0.84) | 0.23 (0.15 - 0.35) | - |
| 2005 | 0.47 (0.37 - 0.59) | 0.8 (0.75 - 0.84) | 0.29 (0.2 - 0.41) | Unreliable |
| 2006 | 0.42 (0.32 - 0.53) | 0.8 (0.75 - 0.84) | 0.21 (0.14 - 0.31) | Unreliable |
| 2007 | 0.36 (0.27 - 0.46) | 0.75 (0.7 - 0.79) | 0.17 (0.11 - 0.27) | Unreliable |
| 2008 | 0.36 (0.28 - 0.46) | 0.75 (0.71 - 0.79) | 0.26 (0.18 - 0.37) | - |
| 2009 | 0.46 (0.37 - 0.57) | 0.8 (0.76 - 0.84) | 0.24 (0.17 - 0.34) | Unreliable |
| 2010 | 0.47 (0.38 - 0.58) | 0.74 (0.7 - 0.78) | 0.25 (0.18 - 0.35) | Unreliable |
| 2011 | 0.48 (0.38 - 0.59) | 0.82 (0.78 - 0.87) | 0.27 (0.19 - 0.36) | Unreliable |
| 2012 | 0.4 (0.32 - 0.5) | 0.77 (0.73 - 0.81) | 0.22 (0.15 - 0.3) | Unreliable |
| 2013 | 0.36 (0.28 - 0.45) | 0.78 (0.74 - 0.82) | 0.19 (0.13 - 0.26) | 0.18 (0.11 - 0.28) |
| 2014 | 0.4 (0.32 - 0.5) | 0.78 (0.74 - 0.82) | 0.23 (0.16 - 0.31) | 0.16 (0.1 - 0.26) |
| 2015 | 0.4 (0.32 - 0.49) | 0.77 (0.73 - 0.81) | 0.23 (0.17 - 0.31) | Unreliable |
| 2016 | 0.42 (0.33 - 0.5) | 0.8 (0.76 - 0.84) | 0.2 (0.14 - 0.28) | 0.17 (0.11 - 0.27) |
| 2017 | 0.44 (0.35 - 0.52) | 0.83 (0.79 - 0.87) | 0.2 (0.14 - 0.27) | 0.22 (0.15 - 0.32) |
| 2018 | 0.43 (0.34 - 0.51) | 0.82 (0.78 - 0.86) | 0.23 (0.17 - 0.3) | 0.19 (0.12 - 0.28) |
| 2019 | 0.5 (0.42 - 0.59) | 0.89 (0.84 - 0.93) | 0.27 (0.21 - 0.34) | 0.21 (0.14 - 0.31) |
| 2020 | 0.7 (0.6 - 0.79) | 1.08 (1.03 - 1.13) | 0.33 (0.26 - 0.41) | 0.31 (0.22 - 0.41) |
| 2021 | 0.58 (0.49 - 0.68) | 1.24 (1.19 - 1.29) | 0.43 (0.35 - 0.51) | 0.22 (0.15 - 0.31) |
| 2022 | 0.77 (0.66 - 0.88) | 1.24 (1.19 - 1.29) | 0.26 (0.21 - 0.33) | 0.3 (0.22 - 0.4) |
| 2023 | 0.64 (0.55 - 0.74) | 1.21 (1.16 - 1.26) | 0.3 (0.24 - 0.38) | 0.22 (0.15 - 0.31) |

**Supplemental Table 5: IBD and CVD-related Age-Adjusted Mortality Rates per 100,000, Stratified by States in the United States, 1999 to 2023**

| State | Age-Adjusted Rate (95% confidence interval) | |
| --- | --- | --- |
|  | **1999-2020** | **2021-2023** |
| Alabama | 0.55 (0.5 - 0.6) | 0.64 (0.5 - 0.8) |
| Alaska | 0.74 (0.54 - 0.99) | Unreliable (0.41 - 1.47) |
| Arizona | 0.5 (0.45 - 0.54) | 0.83 (0.69 - 0.96) |
| Arkansas | 0.55 (0.48 - 0.62) | 0.89 (0.68 - 1.14) |
| California | 0.82 (0.79 - 0.84) | 0.88 (0.82 - 0.94) |
| Colorado | 0.8 (0.73 - 0.87) | 1.55 (1.32 - 1.77) |
| Connecticut | 0.78 (0.7 - 0.85) | 0.68 (0.52 - 0.87) |
| Delaware | 0.5 (0.39 - 0.63) | 0.68 (0.42 - 1.05) |
| District of Columbia | 0.4 (0.28 - 0.55) | - |
| Florida | 0.51 (0.49 - 0.54) | 0.81 (0.74 - 0.88) |
| Georgia | 0.53 (0.49 - 0.58) | 0.84 (0.72 - 0.97) |
| Hawaii | 0.27 (0.2 - 0.34) | - |
| Idaho | 0.85 (0.73 - 0.97) | 1.91 (1.52 - 2.38) |
| Illinois | 0.61 (0.57 - 0.64) | 0.74 (0.64 - 0.84) |
| Indiana | 0.76 (0.7 - 0.81) | 0.99 (0.83 - 1.16) |
| Iowa | 0.72 (0.64 - 0.79) | 1.18 (0.96 - 1.45) |
| Kansas | 0.61 (0.54 - 0.69) | 0.96 (0.74 - 1.24) |
| Kentucky | 0.77 (0.71 - 0.84) | 1.21 (0.99 - 1.42) |
| Louisiana | 0.34 (0.3 - 0.39) | 0.84 (0.68 - 1.04) |
| Maine | 0.84 (0.72 - 0.95) | 1.13 (0.83 - 1.52) |
| Maryland | 0.8 (0.74 - 0.86) | 1.6 (1.39 - 1.81) |
| Massachusetts | 0.78 (0.73 - 0.84) | 0.95 (0.8 - 1.09) |
| Michigan | 0.68 (0.64 - 0.72) | 0.92 (0.8 - 1.03) |
| Minnesota | 0.99 (0.93 - 1.06) | 1.75 (1.53 - 1.97) |
| Mississippi | 0.61 (0.54 - 0.69) | 1.22 (0.97 - 1.52) |
| Missouri | 0.64 (0.59 - 0.69) | 0.74 (0.6 - 0.88) |
| Montana | 0.77 (0.63 - 0.9) | 1.36 (0.97 - 1.86) |
| Nebraska | 1.05 (0.94 - 1.17) | 2.02 (1.63 - 2.49) |
| Nevada | 0.52 (0.44 - 0.59) | 0.92 (0.71 - 1.17) |
| New Hampshire | 0.85 (0.72 - 0.97) | 1.09 (0.79 - 1.48) |
| New Jersey | 0.73 (0.68 - 0.78) | 0.78 (0.66 - 0.89) |
| New Mexico | 0.44 (0.36 - 0.52) | 0.75 (0.53 - 1.02) |
| New York | 0.65 (0.62 - 0.68) | 0.83 (0.75 - 0.9) |
| North Carolina | 0.6 (0.56 - 0.64) | 0.98 (0.85 - 1.11) |
| North Dakota | 0.86 (0.69 - 1.04) | Unreliable (0.55 - 1.55) |
| Ohio | 0.99 (0.94 - 1.03) | 1.2 (1.07 - 1.33) |
| Oklahoma | 1.04 (0.95 - 1.12) | 1.95 (1.66 - 2.24) |
| Oregon | 1.2 (1.11 - 1.29) | 2.09 (1.81 - 2.36) |
| Pennsylvania | 0.72 (0.69 - 0.76) | 1.1 (0.98 - 1.21) |
| Rhode Island | 1.21 (1.06 - 1.37) | 1.53 (1.1 - 2.06) |
| South Carolina | 0.66 (0.6 - 0.72) | 1.14 (0.95 - 1.33) |
| South Dakota | 0.58 (0.46 - 0.72) | 1.04 (0.65 - 1.57) |
| Tennessee | 0.72 (0.67 - 0.78) | 1.13 (0.97 - 1.3) |
| Texas | 0.59 (0.57 - 0.62) | 0.84 (0.76 - 0.92) |
| Utah | 0.72 (0.62 - 0.82) | 1.31 (1.02 - 1.65) |
| Vermont | 1.14 (0.94 - 1.35) | 1.85 (1.26 - 2.61) |
| Virginia | 0.56 (0.51 - 0.6) | 1.01 (0.88 - 1.15) |
| Washington | 0.92 (0.86 - 0.98) | 1.38 (1.21 - 1.56) |
| West Virginia | 0.93 (0.82 - 1.04) | 1.58 (1.22 - 2) |
| Wisconsin | 0.74 (0.68 - 0.8) | 1.3 (1.11 - 1.48) |
| Wyoming | 0.75 (0.57 - 0.96) | 1.94 (1.27 - 2.84) |

**Supplemental Table 6: IBD and CVD-related Age-Adjusted Mortality Rates per 100,000, Stratified by Census Region in the United States, 1999 to 2023**

| Census region | Year | Age-Adjusted Rate (95% confidence interval) |
| --- | --- | --- |
| **Northeast** |  |  |
| Northeast | 1999 | 0.89 (0.79 - 0.98) |
| Northeast | 2000 | 0.8 (0.71 - 0.89) |
| Northeast | 2001 | 0.81 (0.72 - 0.9) |
| Northeast | 2002 | 0.78 (0.7 - 0.87) |
| Northeast | 2003 | 0.73 (0.64 - 0.81) |
| Northeast | 2004 | 0.81 (0.72 - 0.9) |
| Northeast | 2005 | 0.71 (0.63 - 0.79) |
| Northeast | 2006 | 0.75 (0.67 - 0.84) |
| Northeast | 2007 | 0.69 (0.61 - 0.78) |
| Northeast | 2008 | 0.6 (0.52 - 0.67) |
| Northeast | 2009 | 0.79 (0.71 - 0.88) |
| Northeast | 2010 | 0.67 (0.59 - 0.75) |
| Northeast | 2011 | 0.72 (0.64 - 0.8) |
| Northeast | 2012 | 0.69 (0.61 - 0.77) |
| Northeast | 2013 | 0.63 (0.56 - 0.71) |
| Northeast | 2014 | 0.68 (0.6 - 0.76) |
| Northeast | 2015 | 0.65 (0.57 - 0.73) |
| Northeast | 2016 | 0.7 (0.62 - 0.78) |
| Northeast | 2017 | 0.69 (0.61 - 0.77) |
| Northeast | 2018 | 0.71 (0.64 - 0.79) |
| Northeast | 2019 | 0.73 (0.65 - 0.81) |
| Northeast | 2020 | 0.94 (0.85 - 1.03) |
| Northeast | 2021 | 0.98 (0.89 - 1.07) |
| Northeast | 2022 | 0.94 (0.85 - 1.02) |
| Northeast | 2023 | 0.87 (0.79 - 0.95) |
| **Midwest** |  |  |
| Midwest | 1999 | 0.8 (0.72 - 0.89) |
| Midwest | 2000 | 0.74 (0.66 - 0.83) |
| Midwest | 2001 | 0.69 (0.61 - 0.76) |
| Midwest | 2002 | 0.77 (0.68 - 0.85) |
| Midwest | 2003 | 0.83 (0.75 - 0.92) |
| Midwest | 2004 | 0.68 (0.6 - 0.76) |
| Midwest | 2005 | 0.75 (0.67 - 0.84) |
| Midwest | 2006 | 0.84 (0.75 - 0.92) |
| Midwest | 2007 | 0.75 (0.67 - 0.83) |
| Midwest | 2008 | 0.76 (0.68 - 0.84) |
| Midwest | 2009 | 0.75 (0.67 - 0.83) |
| Midwest | 2010 | 0.68 (0.6 - 0.75) |
| Midwest | 2011 | 0.77 (0.7 - 0.85) |
| Midwest | 2012 | 0.69 (0.62 - 0.76) |
| Midwest | 2013 | 0.8 (0.72 - 0.88) |
| Midwest | 2014 | 0.73 (0.66 - 0.81) |
| Midwest | 2015 | 0.72 (0.65 - 0.79) |
| Midwest | 2016 | 0.7 (0.63 - 0.78) |
| Midwest | 2017 | 0.82 (0.74 - 0.9) |
| Midwest | 2018 | 0.71 (0.63 - 0.78) |
| Midwest | 2019 | 0.78 (0.7 - 0.85) |
| Midwest | 2020 | 1.08 (0.99 - 1.16) |
| Midwest | 2021 | 1.12 (1.03 - 1.22) |
| Midwest | 2022 | 1.06 (0.97 - 1.15) |
| Midwest | 2023 | 1.08 (0.99 - 1.17) |
| **South** |  |  |
| South | 1999 | 0.54 (0.48 - 0.6) |
| South | 2000 | 0.59 (0.53 - 0.65) |
| South | 2001 | 0.59 (0.53 - 0.65) |
| South | 2002 | 0.59 (0.53 - 0.65) |
| South | 2003 | 0.58 (0.52 - 0.64) |
| South | 2004 | 0.55 (0.5 - 0.61) |
| South | 2005 | 0.61 (0.55 - 0.67) |
| South | 2006 | 0.57 (0.51 - 0.62) |
| South | 2007 | 0.5 (0.45 - 0.55) |
| South | 2008 | 0.53 (0.47 - 0.58) |
| South | 2009 | 0.57 (0.52 - 0.62) |
| South | 2010 | 0.63 (0.57 - 0.69) |
| South | 2011 | 0.59 (0.54 - 0.65) |
| South | 2012 | 0.6 (0.54 - 0.65) |
| South | 2013 | 0.6 (0.55 - 0.65) |
| South | 2014 | 0.63 (0.57 - 0.68) |
| South | 2015 | 0.56 (0.51 - 0.61) |
| South | 2016 | 0.59 (0.54 - 0.64) |
| South | 2017 | 0.57 (0.52 - 0.62) |
| South | 2018 | 0.62 (0.57 - 0.67) |
| South | 2019 | 0.72 (0.67 - 0.78) |
| South | 2020 | 0.84 (0.78 - 0.9) |
| South | 2021 | 0.95 (0.89 - 1.01) |
| South | 2022 | 1.02 (0.95 - 1.08) |
| South | 2023 | 0.97 (0.91 - 1.03) |
| **West** |  |  |
| West | 1999 | 0.76 (0.67 - 0.85) |
| West | 2000 | 0.68 (0.6 - 0.77) |
| West | 2001 | 0.72 (0.63 - 0.8) |
| West | 2002 | 0.81 (0.72 - 0.9) |
| West | 2003 | 0.81 (0.72 - 0.9) |
| West | 2004 | 0.85 (0.76 - 0.94) |
| West | 2005 | 0.87 (0.78 - 0.97) |
| West | 2006 | 0.74 (0.66 - 0.83) |
| West | 2007 | 0.7 (0.62 - 0.78) |
| West | 2008 | 0.84 (0.75 - 0.93) |
| West | 2009 | 0.78 (0.7 - 0.87) |
| West | 2010 | 0.72 (0.64 - 0.79) |
| West | 2011 | 0.84 (0.76 - 0.93) |
| West | 2012 | 0.74 (0.66 - 0.81) |
| West | 2013 | 0.72 (0.65 - 0.8) |
| West | 2014 | 0.73 (0.66 - 0.81) |
| West | 2015 | 0.72 (0.65 - 0.79) |
| West | 2016 | 0.74 (0.67 - 0.81) |
| West | 2017 | 0.82 (0.74 - 0.9) |
| West | 2018 | 0.74 (0.67 - 0.81) |
| West | 2019 | 0.81 (0.74 - 0.89) |
| West | 2020 | 0.97 (0.89 - 1.05) |
| West | 2021 | 1.13 (1.04 - 1.21) |
| West | 2022 | 1.11 (1.03 - 1.2) |
| West | 2023 | 1.02 (0.94 - 1.1) |

**Supplemental Table 7: IBD and CVD-related Age-Adjusted Mortality Rates per 100,000 in United States stratified by Urban-Rural Classification, 1999-2020**

|  | Age-Adjusted Rate (95% confidence interval) | |
| --- | --- | --- |
| Year | **Urban** | **Rural** |
| 1999 | 0.71 (0.67 - 0.75) | 0.66 (0.57 - 0.75) |
| 2000 | 0.69 (0.65 - 0.73) | 0.67 (0.58 - 0.76) |
| 2001 | 0.68 (0.64 - 0.72) | 0.6 (0.52 - 0.68) |
| 2002 | 0.71 (0.67 - 0.75) | 0.71 (0.62 - 0.8) |
| 2003 | 0.74 (0.69 - 0.78) | 0.68 (0.59 - 0.77) |
| 2004 | 0.7 (0.66 - 0.74) | 0.65 (0.56 - 0.73) |
| 2005 | 0.7 (0.66 - 0.74) | 0.71 (0.62 - 0.79) |
| 2006 | 0.7 (0.65 - 0.74) | 0.74 (0.64 - 0.83) |
| 2007 | 0.65 (0.61 - 0.69) | 0.65 (0.56 - 0.73) |
| 2008 | 0.68 (0.64 - 0.72) | 0.68 (0.6 - 0.77) |
| 2009 | 0.68 (0.64 - 0.72) | 0.74 (0.65 - 0.82) |
| 2010 | 0.66 (0.62 - 0.7) | 0.73 (0.64 - 0.82) |
| 2011 | 0.69 (0.65 - 0.73) | 0.75 (0.66 - 0.84) |
| 2012 | 0.65 (0.62 - 0.69) | 0.75 (0.66 - 0.84) |
| 2013 | 0.66 (0.62 - 0.69) | 0.74 (0.65 - 0.82) |
| 2014 | 0.68 (0.64 - 0.72) | 0.73 (0.65 - 0.82) |
| 2015 | 0.61 (0.58 - 0.65) | 0.76 (0.67 - 0.85) |
| 2016 | 0.68 (0.64 - 0.71) | 0.74 (0.65 - 0.82) |
| 2017 | 0.68 (0.64 - 0.72) | 0.83 (0.73 - 0.92) |
| 2018 | 0.66 (0.63 - 0.7) | 0.78 (0.69 - 0.86) |
| 2019 | 0.76 (0.72 - 0.8) | 0.8 (0.71 - 0.89) |
| 2020 | 0.92 (0.88 - 0.97) | 1.09 (0.99 - 1.2) |

**Supplemental Table 8: Number of deaths and Age-Adjusted Mortality Rates per 100,000 for IBD Related to CVD in which the Underlying Cause of Death Was Restricted to IBD**

| Year | Deaths | Age-Adjusted Rate (95% confidence interval) |
| --- | --- | --- |
| 1999 | 307 | 0.17 (0.15 - 0.2) |
| 2000 | 320 | 0.18 (0.16 - 0.2) |
| 2001 | 316 | 0.17 (0.15 - 0.19) |
| 2002 | 309 | 0.16 (0.14 - 0.18) |
| 2003 | 322 | 0.15 (0.13 - 0.17) |
| 2004 | 344 | 0.17 (0.15 - 0.19) |
| 2005 | 360 | 0.18 (0.16 - 0.2) |
| 2006 | 378 | 0.19 (0.17 - 0.21) |
| 2007 | 327 | 0.17 (0.15 - 0.19) |
| 2008 | 370 | 0.16 (0.14 - 0.17) |
| 2009 | 367 | 0.16 (0.15 - 0.18) |
| 2010 | 317 | 0.11 (0.1 - 0.13) |
| 2011 | 354 | 0.17 (0.15 - 0.18) |
| 2012 | 341 | 0.14 (0.12 - 0.16) |
| 2013 | 374 | 0.14 (0.13 - 0.16) |
| 2014 | 340 | 0.13 (0.11 - 0.14) |
| 2015 | 386 | 0.16 (0.14 - 0.17) |
| 2016 | 372 | 0.14 (0.12 - 0.15) |
| 2017 | 372 | 0.14 (0.13 - 0.16) |
| 2018 | 382 | 0.14 (0.13 - 0.16) |
| 2019 | 442 | 0.15 (0.13 - 0.16) |
| 2020 | 428 | 0.13 (0.12 - 0.15) |
| 2021 | 479 | 0.2 (0.18 - 0.22) |
| 2022 | 512 | 0.18 (0.16 - 0.19) |
| 2023 | 519 | 0.21 (0.19 - 0.23) |

**Supplemental Table 9: Number of deaths and Age-Adjusted Mortality Rates per 100,000 for IBD Related to CVD in which the Underlying Cause of Death Was Restricted to CVD**

| Year | Deaths | Age-Adjusted Rate (95% confidence interval) |
| --- | --- | --- |
| 1999 | 591 | 0.32 (0.3 - 0.35) |
| 2000 | 550 | 0.28 (0.26 - 0.31) |
| 2001 | 519 | 0.29 (0.26 - 0.31) |
| 2002 | 597 | 0.31 (0.28 - 0.33) |
| 2003 | 582 | 0.3 (0.27 - 0.32) |
| 2004 | 548 | 0.27 (0.25 - 0.29) |
| 2005 | 528 | 0.27 (0.24 - 0.29) |
| 2006 | 508 | 0.25 (0.23 - 0.27) |
| 2007 | 492 | 0.24 (0.22 - 0.27) |
| 2008 | 458 | 0.22 (0.2 - 0.24) |
| 2009 | 537 | 0.25 (0.23 - 0.27) |
| 2010 | 502 | 0.22 (0.2 - 0.25) |
| 2011 | 517 | 0.22 (0.2 - 0.24) |
| 2012 | 497 | 0.22 (0.2 - 0.24) |
| 2013 | 463 | 0.2 (0.18 - 0.22) |
| 2014 | 521 | 0.22 (0.2 - 0.23) |
| 2015 | 493 | 0.2 (0.18 - 0.22) |
| 2016 | 526 | 0.23 (0.21 - 0.25) |
| 2017 | 572 | 0.22 (0.2 - 0.24) |
| 2018 | 534 | 0.21 (0.19 - 0.22) |
| 2019 | 590 | 0.22 (0.2 - 0.23) |
| 2020 | 723 | 0.26 (0.24 - 0.28) |
| 2021 | 684 | 0.26 (0.24 - 0.28) |
| 2022 | 823 | 0.29 (0.27 - 0.31) |
| 2023 | 800 | 0.27 (0.25 - 0.29) |
